# Food and Medicine Homology Substances as Potential Modulators of the Gut–Muscle Axis in Animal Meat Quality: A Review

**DOI:** 10.3390/foods15111946

**Published:** 2026-06-01

**Authors:** Zi-Qun Zhang, Fang-Fang Guo, An-Lang Sun, Li Wang, Shu-Cheng Huang

**Affiliations:** 1 College of Veterinary Medicine, Henan Agricultural University, Zhengzhou 450046, China; 18739327974@163.com (Z.-Q.Z.); guofangfang0331@163.com (F.-F.G.); 18337684115@163.com (A.-L.S.); 2 Department of Traditional Chinese Medicine, College of Agronomy, Henan Agricultural University, Zhengzhou 450046, China

**Keywords:** alternatives to antibiotics, functional feed additives, gut microbiota, meat quality, intestinal barrier, skeletal muscle metabolism

## Abstract

Food and medicine homology (FMH) substances are increasingly utilized as nutritional and medicinal resources in sustainable livestock production. Their active ingredients include polysaccharides, flavonoids, and terpenes, which may positively affect livestock meat quality by maintaining gut microbiota homeostasis, enhancing intestinal barrier function, and facilitating nutrient absorption, as well as regulating key signaling pathways such as mechanistic target of rapamycin (mTOR), AMP-activated protein kinase (AMPK), and nuclear factor-κB (NF-κB). Notably, the meat quality improvement can also be indirectly achieved via the gut–muscle axis. Gut microbiota metabolites, including short-chain fatty acids (SCFAs), bile acids (BAs), and amino acid derivatives, modulate microbial homeostasis, intestinal barrier function, and nutrient absorption through the gut microbiota–metabolite axis, gut–immune axis, and nutrient absorption–signaling axis. These processes remotely regulate skeletal muscle metabolism, inflammation, and fiber type transformation, ultimately influencing meat tenderness, flavor, juiciness, and nutritional value. Despite their potential to reduce reliance on antibiotic growth promoters and enhance meat quality, multiple challenges persist, including complex component profiles, elusive mechanisms, undefined dose–effect relationships, inadequate standardization, insufficient safety evaluation and scarce direct trials on livestock meat quality endpoints. This review summarizes FMH substances that modulate the gut–muscle axis in meat quality regulation across different animal species and outlines their application prospects, aiming to facilitate antibiotic-free agriculture, the development of green functional feeds, and sustainable animal husbandry.

## 1. Introduction

The concept of “food and medicine homology” (FMH) originates from traditional Chinese medicine (TCM) and has been documented in China for over three thousand years [1]. This means that edible medicinal materials and conventional foods share similar properties and pharmacological efficacies, allowing them to serve as daily dietary components while exerting therapeutic and health-regulation functions [1,2]. Historically, people commonly combined medicinal plants with meals and focused on maintaining physiological homeostasis through diet to enhance treatment efficacy and nutritional value [3,4]. Application of FMH comes from TCM’s concept of “prevention first”, using daily food to regulate the body and maintain health [4]. Accumulating evidence shows that certain foods can promote satiety and exert diverse biological functions, including health promotion, disease prevention and therapeutic intervention [5]. In human health, TCM has garnered global recognition, contributing to Nobel Prize-winning research and being acknowledged by the World Health Organization as a global medical resource [6]. This trend is mirrored in China’s functional food market, which exceeded 200 billion yuan in 2021, reflecting growing consumer demand for natural and functional products [6]. Today, the livestock industry is adopting similar natural substances to reduce drug reliance and optimize health management, thereby enhancing the sustainability of production practices [7]. Compared with conventional antibiotic-reliant farming, FMH substances offer a healthier and greener production, minimizing chemical residue and drug-associated toxicity; moreover, these natural substances confer multiple superior properties such as multi-targeting bioactivities and biosafety [7,8]. Therefore, FMH substances possess broad potential as safe alternatives to antibiotics in modern livestock and poultry production.

Modern intensive livestock production effectively satisfies global meat demand, yet high-efficiency rearing systems often induce metabolic stress and compromised meat quality [9,10]. Meat quality is a multifaceted commercial and physiological trait assessed by standardized measurable endpoints, including pH decline, meat color, water-holding capacity, drip loss, cooking loss, shear force, tenderness, intramuscular fat (IMF) content, flavor volatiles, oxidative stability, and shelf-life [7,9,10]. Antibiotic overuse disrupts gut microbiota homeostasis and interferes with animal metabolic and inflammatory status, thereby impairing meat sensory characteristics and nutritional properties, while antibiotic residues mainly pose food safety and regulatory risks [7]. Notably, antibiotic-induced gut dysbiosis is recognized as a key driver of disturbed muscle metabolism and altered muscle fiber characteristics, ultimately deteriorating meat quality [11,12]. In this context, the gut–muscle axis is defined as a bidirectional communication network between gut microbiota and skeletal muscle via metabolic, immune and endocrine signaling pathways, and relevant studies in rabbits have validated its pivotal role in regulating muscle growth and development [11,12]. This axis can be strategically modulated; for instance, dietary supplementation with short-chain fatty acids (SCFAs) or specific amino acids can reshape gut microbiota composition, while targeted probiotics can facilitate the dominance of beneficial intestinal bacteria, thereby regulating muscle function directly or indirectly [11,13].

Although existing studies confirm the positive effects of various FMH substances on livestock intestinal health and meat quality, these findings remain scattered and fragmented [14,15]. Most previous reviews only focus on the growth-promoting or antioxidant functions of FMH substances or simply summarize their individual application effects in animal production, and generally fail to distinguish FMH substances from conventional phytogenic additives [14,15,16]. Distinct from conventional plant extracts, essential oils, probiotics, prebiotics and other non-FMH natural additives, FMH substances embody dual nutritional and medicinal properties, accompanied by favorable biosafety and multi-target regulatory effects [17]. Furthermore, the recent literature predominantly emphasizes the roles of these substances in maintaining general intestinal health, rarely adopting the gut–muscle axis as a necessary theoretical framework to elucidate meat quality formation [18,19]. There is still a lack of holistic and targeted comprehensive reviews that integrate the regulatory mechanism of FMH substances on meat quality based on the gut–muscle axis perspective. The intrinsic relationship between FMH substances, gut microbiota and meat quality formation remains poorly clarified, leaving the core regulatory mechanism by which FMH substances modulate livestock meat quality incompletely understood. To bridge this gap, we propose that FMH substances can serve as key regulators of the gut–muscle axis. By remodeling gut microbiota composition, modulating microbial metabolite signals, maintaining intestinal immune homeostasis, and optimizing nutrient absorption, FMH substances remotely regulate skeletal muscle physiology and metabolism, ultimately improving meat quality in livestock and poultry [11]. This review summarizes the active ingredients and physiological functions of common FMH substances, elucidates their gut–muscle axis-mediated mechanisms in regulating meat quality, and evaluates their application efficacy and research progress in various animal models, aiming to provide new insights for the development and application of FMH-based strategies in sustainable livestock and poultry production.

## 2. FMH Classification and Bioactivities

**Figure 1:**
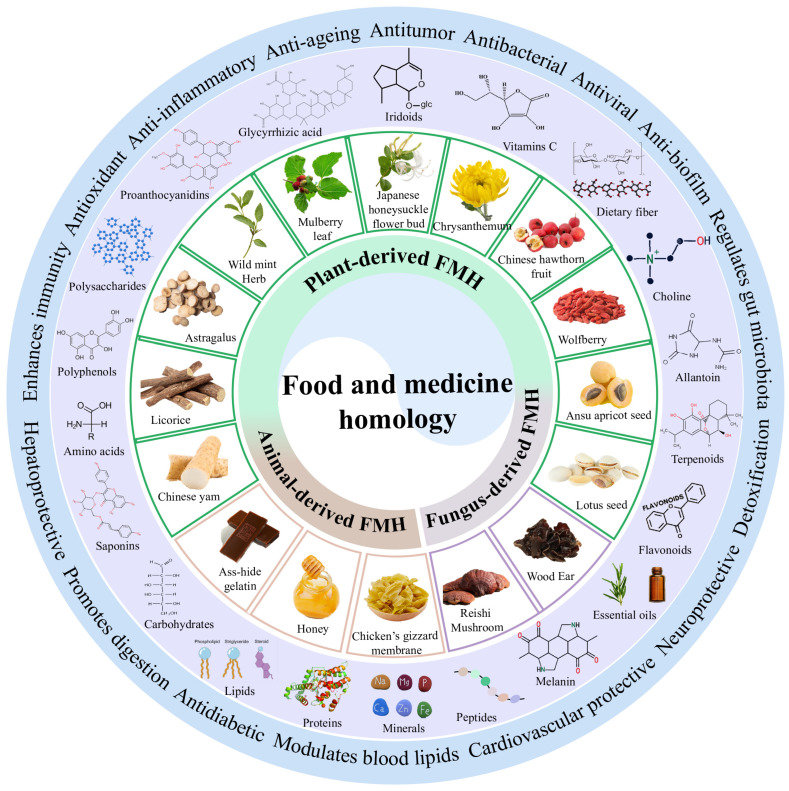
Source classification, bioactive components and functions of common FMH substances. FMH substances are primarily derived from plant, animal, and fungal sources. This diagram outlines key bioactive components they contain, including polysaccharides, phenolics, flavonoids, terpenoids, and alkaloids, along with their pharmacological effects.

FMH has been practiced for centuries in TCM, where it is recognized for its nutritional and health-promoting properties [2]. These attributes have garnered increasing interest in both food science and medical research [20]. In daily life, FMH substances are widely present in common foods, which can meet basic nutritional requirements as well as exert health-promoting effects, consistent with the preventive healthcare philosophy of TCM [6]. Depending on their source, FMH substances can be classified into plant-derived, animal-derived, and fungus-derived types [5,6]. Figure 1 illustrates the overall classification, major bioactive components and representative functions of FMH substances at the macro level, while Table 1 provides detailed TCM properties, specific pharmacological effects and corresponding references of typical FMH candidates. The health-promoting effects of these FMH substances ultimately stem from their specific chemical constituents. To further elucidate their underlying mechanisms, it is essential to evaluate their bioactive components from a modern scientific perspective.

### 2.1. Plant-Derived FMH

#### 2.1.1. Rhizomes and Roots

Plant materials contain abundant bioactive compounds and essential nutrients, including vitamins, minerals, dietary fiber, amino acids, and unsaturated fatty acids [5,6]. Major rhizome- and root-derived FMH raw materials include Dioscoreae Rhizoma (Chinese yam), Glycyrrhizae Radix et Rhizoma (licorice), Astragali Radix (astragalus), Panax Ginseng Radix (ginseng), Allii Sativi Bulbus (garlic), Zingiberis Rhizoma (ginger), Polygonati Rhizoma (polygonatum), and Puerariae Lobatae Radix (kudzu root). Widely distributed and naturally abundant, these plant resources have attracted increasing attention owing to their active constituents such as flavonoids, polysaccharides, and alkaloids, which exert potent antioxidant and anti-inflammatory activities [17,83,84].

Traditionally, Chinese yam is used to regulate spleen and stomach function, promote fluid production, moisten the respiratory tract, and modulate renal physiological function [6]. Allantoin, fatty acids, amino acids, proteins and non-starch polysaccharides endow Chinese yam with multiple biological functions, among which its polysaccharides exhibit prominent immunomodulatory, antioxidant, anti-aging, anti-tumor, hypoglycemic and gastrointestinal protective activities [21,22,23,24,25,26]. Licorice originates from the root and rhizome of *Glycyrrhiza* species [28], and its primary bioactive components, including glycyrrhizic acid, glycyrrhetinic acid, and flavonoids, exhibit anti-inflammatory, antioxidant, antiviral, and immunomodulatory properties; glycyrrhizic acid is metabolized by gut microbiota into glycyrrhetinic acid, which accounts for most of licorice’s pharmacological and antiviral effects [27,29,30]. Triterpenoid saponins, polysaccharides and flavonoids in astragalus exert immunoregulatory, hypoglycemic, antioxidant, anti-inflammatory, antiviral and anti-tumor activities [31]; meanwhile, astragalus also provides cardioprotective benefits and adjuvant effects in diabetic management with good safety, and its polysaccharides play a central role in modulating immune function, tumor progression and gut microbiota composition [31,85].

#### 2.1.2. Leaves

Leaf-derived FMH differ from rhizome and root materials in chemical composition, being rich in secondary metabolites such as polyphenols, volatile oils, and alkaloids, which contribute to distinct flavor characteristics and pharmacological properties [34]. Common leaf-based FMH include Menthae Haplocalycis Herba (wild mint herb), Mori Folium (mulberry leaf), Nelumbinis Folium (Hindu lotus leaf), Lophatheri Herba (common lophatherum herb), and Perillae Folium (Perilla leaf).

For instance, the characteristic aroma and bioactivities of wild mint herb are attributed to its flavonoids, phenolic lignans, stilbenes, and essential oils, which provide antioxidant, antibacterial, anti-inflammatory, and gastrointestinal regulatory effects, supporting its traditional application value [32,33,34]. Mulberry leaf contains abundant polysaccharides, phenols and nutritional components, which endow it with hypoglycemic, free radical scavenging, and anti-inflammatory abilities [36,37,41]. Meanwhile, its enzymatic hydrolysates and flavonoids can enhance antioxidant enzyme activity, reduce oxidative stress, and protect cell membrane integrity [35,38,39,40].

#### 2.1.3. Flowers

Major flower-derived FMH materials include Lonicerae Japonicae Flos (honeysuckle flower bud), Chrysanthemi Flos (chrysanthemum), Osmanthi Flos (sweet osmanthus), Sophorae Flos (pagoda tree flower), and Rosae Rugosae Flos (rose flower). Besides ornamental value, these flowers contain diverse, unique bioactive components and are widely utilized in TCM and modern healthcare products [48].

Among them, Lonicerae Japonicae Flos contains iridoids, organic acids, flavonoids and polysaccharides, which exert anti-inflammatory, antibacterial, antioxidant and immunomodulatory activities [42,45]; its polysaccharides also exhibit anti-diabetic, anti-tumor and free radical-scavenging capacities by regulating downstream signaling pathways [43,44]. Chrysanthemum is rich in flavonoids, caffeoylquinic acids and terpenoids, which endow it with prominent antioxidant, anti-inflammatory and neuroprotective properties [46,47,48,49].

#### 2.1.4. Fruits

Fruit-derived FMH are commonly consumed edible functional materials, mainly including Lycii Fructus (wolfberry), Crataegi Fructus (Chinese hawthorn), Jujubae Fructus (jujube), and Mori Fructus (mulberry fruit). They are rich in vitamins, organic acids, dietary fiber, polysaccharides, flavonoids and anthocyanins, providing nutritional supplementation, free radical scavenging, immune enhancement, digestive health improvement and lipid regulation effects [50,53,55].

For example, wolfberry is abundant in essential amino acids (EAAs), unsaturated fatty acids, vitamin C and minerals, which endow it with immunomodulatory, antioxidant, anti-tumor, anti-inflammatory and hepatorenal protective effects [50,51,52]. Chinese hawthorn fruit is rich in polyphenols, flavonoids, pectin and vitamin C, which endow it with digestive-promoting, antioxidant, anti-inflammatory and cardiovascular protective properties [53,56]. Its leaves and flowers are also high in polyphenols, with their antioxidant activity closely associated with flavan-3-ol composition [53,56].

#### 2.1.5. Seeds

Seed-derived FMH materials include Armeniacae Semen Amarum (ansu apricot seed), Nelumbinis Semen (lotus seed), Sesami Semen (sesame seed), Coicis Semen (coix seed), and Cassiae Semen (cassia seed). These natural resources are widely consumed as food and TCM health-regulating materials, rich in unsaturated lipids, proteins, vitamin E, minerals, dietary fiber, flavonoids, saponins and alkaloids [59,63]. They generally exhibit antioxidant, anti-inflammatory, lipid-regulating and gut health-promoting properties.

Ansu apricot seed, which has high protein and lipid contents and has long been used in diet and healthcare, is abundant in vitamin E, lipids and polyphenols that exert antioxidant, antibacterial and anti-inflammatory activities, with compound fermentation further enhancing these biofunctions [57]. Its bioactive peptides can alleviate UV-induced damage and skin aging [59], while network pharmacology evidence reveals that bitter apricot seed relieves pulmonary inflammation by suppressing inflammatory factor release via the regulation of macrophage activity and the Caspase-3 pathway [58]. Lotus seed, serving as a traditional edible functional material with a long application history, contains proanthocyanidins, flavonoids, alkaloids and amino acids, which endow it with potent antioxidant, anti-inflammatory, anti-tumor and metabolic regulatory capacities [60,61,62,63].

### 2.2. Animal-Derived FMH

As an important category of FMH substances, animal-derived functional materials contain abundant bioactive peptides, collagen and phenolic components with conserved antioxidant, anti-inflammatory and gut microbiota-modulating activities [86,87]. Although most of them are traditionally applied for human medicinal purposes, their key active ingredients and physiological regulatory mechanisms provide important theoretical references for screening natural feed additives, alleviating livestock oxidative stress and inflammatory injury, and further improving meat quality via the gut–muscle axis [86,87]. Typical representatives include natural honey (Mel), ass-hide gelatin (Asini Corii Colla) and chicken’s gizzard membrane (Galli Gigerii Endothelium Corneum), all of which are rich in collagen, bioactive peptides and enzymes and exert multiple physiological regulatory properties [74,86,87].

For instance, natural honey consists primarily of glucose, fructose, carbohydrates, trace proteins and amino acids, among which phenolic compounds serve as its key antioxidant constituents [65,69]. Rich in phenolic acids and flavonoids, honey exerts prominent antibacterial, anti-inflammatory and antioxidant effects [64,66,67,68], and it can regulate blood lipids and inflammatory factors by lowering serum cholesterol, triglycerides and tumor necrosis factor-alpha (TNF-α) while upregulating high-density lipoprotein and antioxidant enzyme levels; additionally, it modulates gut microbiota structure and reduces serum free fatty acid accumulation to maintain physiological homeostasis [70]. Ass-hide gelatin (E’jiao) is mainly composed of collagen hydrolysate, trace elements and glycosaminoglycan [72]. Its fractions with different molecular weights exhibit excellent antioxidant and immunomodulatory activities, enhance the phagocytic ability of RAW264.7 cells, and modulate the secretion of nitric oxide, reactive oxygen species, TNF-α and interleukin (IL)-6 [73]. Moreover, E’jiao possesses strong antibacterial efficacy against *Salmonella typhimurium* in both in vitro and in vivo models [71]. Overall, E’jiao possesses remarkable immunoregulatory and antimicrobial properties, which can serve as a promising natural functional substance for regulating livestock physiological health and improving meat quality.

### 2.3. Fungus-Derived FMH

Edible fungi have long been utilized as nutritional and medicinal resources in China. Representative fungus-derived FMH include Ganoderma Lucidum (reishi mushroom), Auricularia (wood ear), Ophiocordyceps Sinensis (caterpillar fungus), and Wolfiporia Cocos (poria cocos) [88]. These fungi are traditionally applied to improve blood circulation, enhance bodily defense, reduce internal heat and relieve mass nodules [88]. They are rich in polysaccharides, phenols, triterpenes and sterols, exerting immunoregulatory, anti-tumor, antiviral, neuroprotective and metabolic regulatory activities [89,90,91]. The broad bioactivities of edible fungi make them promising natural candidates for functional food development.

Reishi mushroom (Ganoderma Lucidum) contains major bioactive constituents, including polysaccharides and ganoderic acid-type triterpenoids, which confer it with anti-tumor, antioxidant, anti-inflammatory and immunomodulatory properties [8,76,77]. Its polysaccharides can regulate gut microbiota and immune function through multiple targets and signaling pathways, supporting its development as a natural health-promoting functional raw material [8]. Wood ear mushroom, with a long history of food and medicinal use in China, is rich in polysaccharides, melanin and phenolic acids that endow it with great research value as a functional food [79,80,82]. Distinct from other edible fungi, its abundant melanin exhibits potent antioxidant, anti-biofilm and hepatoprotective activities [78,81].

## 3. Mechanisms of the Gut–Muscle Axis Regulating Meat Quality

### 3.1. Gut Microbiota–Metabolite Axis

It is critical to differentiate in vivo muscle physiological regulation mediated by the gut–muscle axis from post-slaughter meat quality formation. Through microbial metabolites such as SCFAs, the gut–muscle axis indirectly affects meat quality by regulating myofiber development in live livestock [11]. However, beyond this in vivo regulation, final postmortem meat quality is determined by a complex interplay of factors, including slaughter stress, glycogen reserves, postmortem pH decline, proteolysis, connective tissue characteristics, oxidation status and water distribution [10,92]. Therefore, the gut–muscle signaling pathway merely acts as one influential regulatory branch, rather than the predominant factor governing overall meat quality traits.

**Figure 2:**
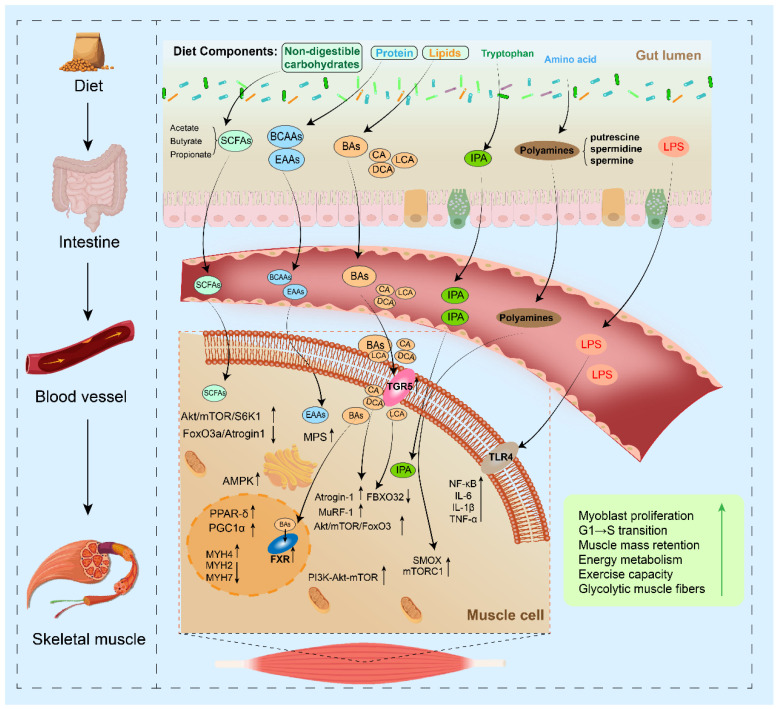
The gut microbiota–metabolite axis modulates muscle quality. SCFAs, short-chain fatty acids; BCAAs, branched-chain amino acids; EAAs, essential amino acids; BAs, bile acids; CA, cholic acid; LCA, lithocholic acid; DCA, deoxycholic acid; IPA, indole-3-propionic acid; LPS, lipopolysaccharides; TGR5, G protein-coupled bile acid receptor 1 (also known as PBAR1); TLR4, Toll-like receptor 4; NF-κB, nuclear factor kappa-B; IL, interleukin; TNF-α, tumor necrosis factor-α; SMOX, spermine oxidase; mTORC1, mechanistic target of rapamycin complex 1; MuRF-1, muscle RING-finger protein-1; Akt, Protein Kinase B; mTOR, mechanistic target of rapamycin; Atrogin-1, F-box only protein 32 (also known as FBXO32); FoxO3, Forkhead box O3; S6K1, p70 ribosomal protein S6 kinase 1; FXR, farnesoid X receptor; AMPK, AMP-activated protein kinase; PPAR-δ, peroxisome proliferator-activated receptor delta; PGC1α, peroxisome proliferator-activated receptor gamma coactivator 1-alpha; MYH, myosin heavy chain.

The gut microbiota generates a variety of structural components and metabolites that facilitate bidirectional communication between the intestine and muscle [93,94]. These include microbial structural components like lipopolysaccharides (LPS), as well as key metabolites such as SCFAs, bile acids (BAs) and tryptophan derivatives [93,94,95]. Microbial metabolites act as a link between gut microbiota and skeletal muscle [94], which regulates muscle function by modulating systemic and tissue-level physiological processes, and by regulating insulin sensitivity in muscle, as shown in Figure 2 [96]. Gut microbiota ferment dietary fibers and conduct proteolytic metabolism of protein substrates to produce intermediate metabolites such as SCFAs, which enter the circulation, enhance insulin sensitivity, and regulate inflammatory pathways, thereby promoting mitochondrial biogenesis in muscle cells and improving energy supply capacity [97]. Despite the anatomical isolation of the gut and skeletal muscle, changes in the composition of gut microbiota can still enable so-called “long-distance” communication via metabolite intermediaries like SCFAs and amino acid derivatives [96]. These metabolites enter the bloodstream and act as carriers that regulate metabolism in distant tissues, which underpins the concept of the “gut–muscle axis” [96].

SCFAs, primarily fermented from undigested carbohydrates by gut microbiota [98], act as pivotal regulators in the gut–muscle axis. After intestinal absorption and entry into the circulation, acetate, propionate, and butyrate modulate skeletal muscle metabolism either by directly binding to SCFA receptors expressed in muscle tissue or indirectly through endocrine, immune, hepatic and neural pathways [94,99]. Functionally, SCFAs regulate muscle energy homeostasis by activating AMP-activated protein kinase (AMPK), which further modulates the expression and activity of peroxisome proliferator-activated receptor gamma coactivator 1-alpha (PGC1α); specifically, acetate and propionate enhance insulin-independent glucose uptake in L6 and C2C12 myotubes, while butyrate upregulates PPAR-δ to facilitate mitochondrial respiration [94,100]. This coordinated signaling activation enables SCFAs to modulate skeletal muscle energy metabolism and physiological performance [94].

Muscle mass is governed by the dynamic balance between protein synthesis and degradation, which are modulated by nutrition, hormonal status, physical activity, and pathological conditions [101]. SCFAs regulate lipid, carbohydrate and protein metabolism in skeletal muscle both in vitro and in vivo, thereby preserving muscle mass, peripheral blood flow, insulin sensitivity, and the oxidative phenotype [94]. Clinical evidence has linked reduced abundances of Firmicutes and Proteobacteria with lower acetate and butyrate levels, while in vitro data indicate that butyrate facilitates the G1/S cell cycle transition and enhances C2C12 myocyte proliferation [102]. In murine models, SCFAs promote myotube growth and muscle accretion by inhibiting the Forkhead box O3a (FoxO3a)/Atrogin1-mediated protein degradation pathway and activating the protein kinase B/mammalian target of rapamycin/ribosomal S6 kinase 1 (Akt/mTOR/S6K1) anabolic cascade, with mTOR signaling serving as a central mediator of these beneficial effects [103]. Collectively, these preclinical and in vitro findings illustrate potential mechanistic pathways for muscle metabolic regulation. These lines of evidence can serve as mechanistic background and indirect reference, suggesting that physiological SCFA supplementation could serve as a promising nutritional strategy to maintain muscle homeostasis, which further provides indirect mechanistic implications for meat quality improvement [103].

Dietary protein-derived amino acids serve as primary regulators of postprandial muscle protein synthesis and remodeling to sustain muscle mass [104], while EAAs modulate myofibrillar protein synthesis in healthy humans [105]. Amino acid metabolism is tightly linked to muscle homeostasis, given that skeletal muscle is protein-dominant and its turnover relies largely on amino acid availability [106]. Impaired branched-chain amino acid (BCAA) catabolism, a common feature in sarcopenia, induces BCAA accumulation and exacerbates muscle dysfunction by dysregulating mTOR signaling; accordingly, pharmacological enhancement of BCAA catabolism via the BCKDK allosteric inhibitor BT2 protects aged and Ppm1k knockout mice from muscle atrophy [106]. Certain gut microbiota utilize dietary BCAAs and reduce host BCAA bioavailability for muscle protein synthesis, thereby compromising muscle mass [107]. Gut dysbiosis further enriches BCAA-degrading taxa, limiting the BCAA supply for muscle maintenance, whereas gut microbiota also synthesize multiple EAAs to sustain systemic amino acid homeostasis [108]. Enrichment of SCFA-producing microbes can alleviate BCAA depletion, maintain bile acid homeostasis and secondary bile acid generation, and potentially protect against muscle wasting [108].

BAs are steroid-derived signaling molecules that mediate digestion, metabolism and immune homeostasis, and they bidirectionally modulate muscle atrophy and regeneration via receptor signaling and gut microbiota interaction, potentially linking them to meat quality regulation [108]. Primary BAs are synthesized by the host, whereas secondary BAs are transformed from primary counterparts by gut microbiota [109]. A balanced bile acid profile facilitates muscle anabolism and attenuates muscle catabolism [108,110]. Altered bile acid metabolism has been observed in cancer cachexia models, where elevated total bile acid levels coincide with reduced gastrocnemius mass and apparent muscle atrophy [110]. BAs modulate the balance of skeletal muscle protein synthesis and degradation by activating the G protein-coupled bile acid receptor 1 (GPBAR1, also called TGR5) and the farnesoid X receptor (FXR) [111]. Cholic acid (CA) and deoxycholic acid (DCA) facilitate muscle atrophy via the TGR5 pathway, as evidenced by reduced muscle fiber size, decreased myosin heavy chain expression, upregulated atrophy-related genes (Atrogin-1 and MuRF-1), and elevated reactive oxygen species and autophagy levels [111]. In contrast, lithocholic acid (LCA) acts through TGR5 to activate the Akt/mTOR/FoxO3 cascade, which suppresses the atrophy-related protein F-box only protein 32 and promotes myocyte differentiation and regeneration to alleviate muscle injury [112]. Gut microbiota regulate muscle function by converting primary BAs into secondary metabolites such as DCA and LCA; thus, gut dysbiosis alters secondary bile acid profiles, potentially inducing muscle atrophy through FXR or TGR5 signaling [113]. Exercise remodels gut microbiota composition to enhance BA deconjugation, thereby activating muscular AMP-activated protein kinase (AMPK) and improving glucose uptake and energy metabolism [114]. In livestock, colonic bile acid profiles are shaped by host–microbiota interactions. Specifically, dihydrocholic acid (DHCA) contributes to meat quality regulation by modulating muscle fiber development and fatty acid deposition, while glycocholic acid (GCA) primarily affects carcass characteristics in ruminants [93]. Furthermore, interventions like nobiletin upregulate hepatic cholesterol 7-alpha hydroxylase to modulate bile acid synthesis and microbial bile acid metabolism, while downregulating muscular atrophy proteins (TRIM63 and F-box only protein 32) and activating the Akt/mTOR pathway, thereby attenuating high-fat diet-induced muscle loss and metabolic dysfunction [115].

Beyond the microbial metabolites discussed above, tryptophan-derived indoles, microbial or host-derived polyamines, and LPS-mediated inflammatory signaling also exert regulatory effects on muscle properties. Tryptophan is microbially converted into indole-3-propionic acid (IPA), which modulates muscle mass and muscle fiber phenotype by upregulating MYH4 while suppressing the slow-fiber markers MYH7 and MYH2, thereby elevating the proportion of glycolytic muscle fibers and altering muscle contractile characteristics [116]. IPA also improves insulin sensitivity and activates the phosphatidylinositol 3-kinase (PI3K)/Akt/mTOR cascade to facilitate muscle protein synthesis and myogenic differentiation; in vitro evidence further confirms that IPA directly promotes C2C12 myoblast differentiation, verifying its pivotal role in modulating muscle cell growth [116]. These in vivo findings on muscle physiology may serve as potential regulatory mechanisms for meat quality, but the relationship between glycolytic fiber proportion and meat quality requires further investigation.

LPS, a component of the outer membrane of Gram-negative bacteria, binds to Toll-like receptor 4 (TLR4) on skeletal muscle cell membranes, triggering the nuclear factor kappa-B (NF-κB) signaling pathway and upregulating pro-inflammatory cytokines, including IL-6, IL-1β, and TNF-α [117,118]. These cytokines induce the expression of muscle atrophy-related genes, muscle RING-finger protein-1 (MuRF1) and MAFbx, accelerate muscle protein degradation, and ultimately reduce muscle mass [117,118]. Conversely, the probiotic metabolite butyrate reduces LPS leakage by enhancing intestinal barrier function and relieves muscle inflammation by promoting M1-to-M2 macrophage polarization [119]. Butyrate also alleviates sarcopenia-related muscle atrophy by regulating the Akt/mTOR/FoxO3a and Fbox32/Trim63 pathways [119].

Polyamines rely on ornithine decarboxylase and S-adenosylmethionine decarboxylase for endogenous biosynthesis in host muscle cells, and employ enzymes such as N-acyltransferase, polyamine oxidase and spermine oxidase (SMOX) to maintain catabolic homeostasis [120]. The rate-limiting enzymes for polyamine synthesis are regulated via the androgen receptor, through which androgens facilitate muscle growth [121]. Polyamines activate anabolic signaling to induce muscle hypertrophy, and SMOX expression is upregulated during muscle development; SMOX overexpression increases myofiber diameter, whereas its inhibition reduces myofiber thickness [120]. Mechanistically, polyamines modulate protein synthesis mainly through the mechanistic target of rapamycin complex 1 (mTORC1) pathway [122]. Mechanical stimulation-induced muscle growth elevates the expression of ornithine decarboxylase 1 (ODC1) and spermidine synthetase, and this effect is abrogated by the mTORC1 inhibitor rapamycin, indicating that polyamine metabolism coordinates with mTORC1 activity to regulate muscle mass [122].

In summary, microbial metabolites, including SCFAs, bile acids and amino acid derivatives, construct a gut–muscle regulatory network via immune, metabolic and neuroendocrine pathways [93,94,95]. The gut microbiota interacts with skeletal muscle mainly through these metabolites, offering promising targets for intervening in metabolic disorders and muscle dysfunction [107,108,109]. Further elucidation of the underlying mechanisms of microbial metabolites in muscle tissue will provide novel strategies to modulate muscle quality in livestock via targeted gut microbiota regulation.

### 3.2. Gut–Immune Axis

**Figure 3:**
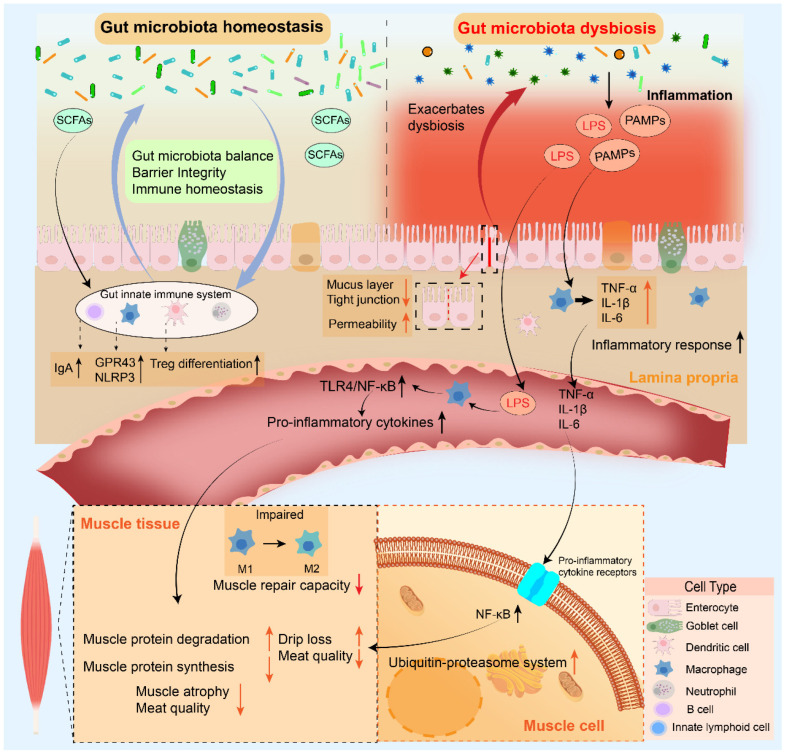
The intestinal mucosal immune axis modulates meat quality. SCFAs, short-chain fatty acids; LPS, lipopolysaccharides; PAMPs, pathogen-associated molecular patterns; IL, interleukin; TNF-α, tumor necrosis factor-α; IgA, immunoglobulin A; GPR43, G protein-coupled receptor 43; NLRP3, NOD-like receptor family pyrin domain-containing 3; TLR4, Toll-like receptor 4; NF-κB, nuclear factor kappa-B.

The intestinal mucosal immune system, as one of the largest immune interfaces in the body, modulates systemic inflammation through multiple pathways [123,124,125,126]. Impaired intestinal barrier facilitates the translocation of pathogen-associated molecular patterns (PAMPs) such as endotoxins, which activate immune cells in the intestinal lamina propria and trigger local and systemic low-grade inflammation, thereby further influencing muscle tissue (Figure 3) [127,128]. The gut–immune axis serves as a critical regulatory hub, wherein inflammatory mediators, including TNF-α, IL-1β and IL-6, act as core messengers to modulate muscle damage and repair, thus potentially influencing muscle quality [124,128]. Persistent inflammation impairs skeletal muscle performance and post-injury repair capacity, representing a major driver of muscle function decline [129,130]. Chronic inflammation also compromises skeletal muscle regeneration, in which macrophages exert indispensable roles; dysregulated M1/M2 macrophage polarization disrupts satellite cell activation and fibroblast adipogenic progenitor cells’ survival, thereby weakening muscle injury repair [129]. Cytokines, as key immune regulatory molecules, play a central role in this process. Inflammatory factors such as TNF-α may alter muscle cell membrane permeability and facilitate intracellular fluid leakage by activating the NF-κB pathway, which could potentially be associated with postmortem muscle drip loss and meat quality [124,131].

Gut microbiota dysbiosis, commonly referred to as intestinal imbalance, is frequently observed in various diseases such as inflammatory bowel disease and pathogenic microbial infections [125,132]. Disruption of the gut microbiota and the invasion of exogenous harmful substances can lead to intestinal barrier damage, resulting in increased secretion of inflammatory cytokines that subsequently impair muscle growth and development [130]. For example, in inflammatory bowel disease, elevated levels of pro-inflammatory cytokines increase the inflammatory load and severely disrupt cell-to-cell adhering structures, leading to the disruption of the intestinal epithelial barrier [125]. The damaged intestine further aggravates microbial imbalance through heightened inflammatory reactions and increased intestinal permeability [125]. At the same time, the gut microbiota is dysbiotic and decreases the abundance and diversity of commensal bacteria, disrupts the mucus layer, thus allowing invasive pathogens to access intestinal epithelial cells and trigger a series of immune reactions [127,128,130]. Alterations in intestinal epithelial barrier permeability may promote the translocation of LPS into the systemic circulation [130]. PAMPs, such as LPS, enter the bloodstream and activate the TLR4/NF-κB signaling pathway, inducing the release of pro-inflammatory cytokines and triggering systemic inflammation [127,128,130,133]. Inflammatory mediators carried through the blood into the muscles can activate the ubiquitin-proteasome system (UPS) to accelerate muscle protein breakdown and inhibit muscle growth, which may theoretically influence muscle atrophy and postmortem meat quality [127,130,133].

Furthermore, the gut microbiota and its metabolites such as SCFAs, along with antigenic stimuli, participate in the development and function of the intestinal mucosal immune system [134]. The interactions among gut microbiota, epithelial cells, macrophages, dendritic cells, neutrophils, and innate lymphoid cells preserve microbial balance, maintain intestinal mucosal barrier integrity, and promote immune homeostasis via the gut innate immune system [135]. This local immunoregulation triggers systemic responses and serves as a core mechanism to restrain excessive inflammation and sustain immune homeostasis [135]. As key immune homeostatic regulators, SCFAs maintain immune homeostasis by binding to receptors such as G protein-coupled receptor 43 (GPR43) and activating the NOD-like receptor family pyrin domain-containing 3 (NLRP3) inflammasome [136]. In the context of intestinal epithelial homeostasis, this regulated activation facilitates IL-18 release, accelerates intestinal epithelial repair, and enhances intestinal barrier integrity [136,137]. These processes not only prevent local colitis but also mediate systemic immunoregulation via the suppression of overactive inflammation [136,137]. SCFAs also increase intestinal IgA, support B-cell development, and play an essential role in promoting the differentiation and expansion of regulatory T cells (Tregs) [136,138]. Commensal microbiota promote Treg differentiation and help maintain immune tolerance [139]. Conversely, dysbiosis, such as the enrichment of pathogenic bacteria, can activate Th17 cells and pro-inflammatory innate lymphoid cells, releasing inflammatory factors such as IL-6 and TNF-α [125,140]. These cytokines disrupt intestinal barrier integrity, allowing LPS and pathogenic bacteria to enter the bloodstream and tissue, which can cause problems such as redness and pain in the intestines, as well as local inflammation [125,141]. A study performed on mice demonstrated that fecal microbiota from young mice transplanted into aged mice reduced LPS levels, thereby inhibiting the TLR4/NF-κB signaling pathway and subsequent production of pro-inflammatory mediators [133]. Gut microbiota modulate the immune–inflammatory axis bidirectionally through metabolites such as SCFAs and BAs, as well as immune cells such as T cells and macrophages [125,133]. Therefore, regulating gut microbiota to optimize SCFA metabolism and targeting the LPS/TLR4 pathway to intervene in the gut–muscle axis may emerge as a novel strategy to improve gut immune homeostasis, alleviate inflammation, and ultimately enhance muscle physiology and meat quality [125,133,141].

### 3.3. Nutrient Absorption Signaling Axis

**Figure 4:**
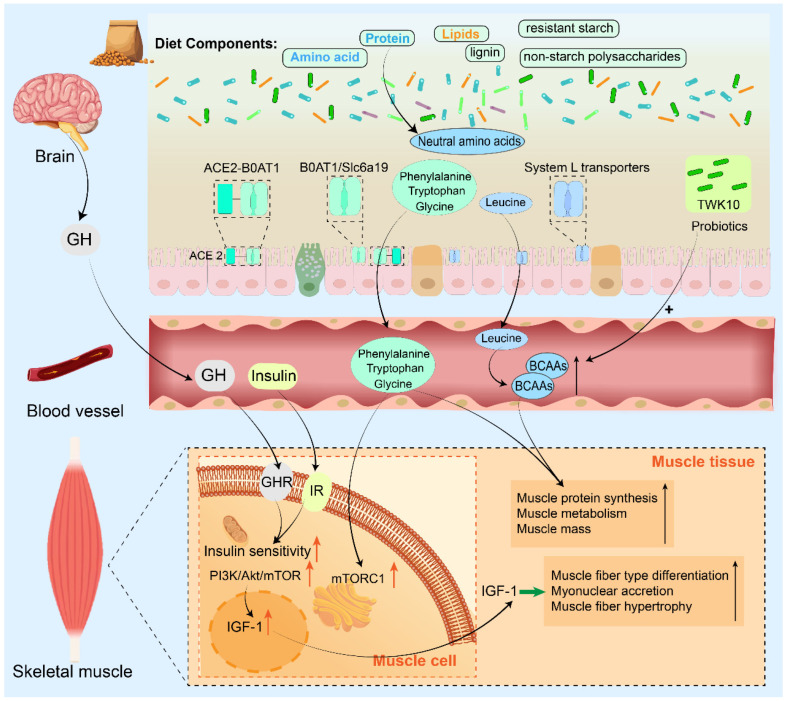
The intestinal nutrient absorption-signaling axis modulates meat quality. BCAAs, branched-chain amino acids; TWK10, TWK10 probiotic; GH, growth hormone; GHR, growth hormone receptor; IR, insulin receptor; PI3K, phosphatidylinositol 3-kinase; Akt, Protein Kinase B; mTOR, mechanistic target of rapamycin; mTORC1, mechanistic target of rapamycin complex 1; IGF-1, insulin-like growth factor 1; ACE2, angiotensin-converting enzyme 2; B0AT1, neutral amino acid transporter 1; Slc6a19, solute carrier family 6 member 19.

As a key organ for nutrient absorption in livestock, the intestine assimilates most dietary nutrients [135]. The efficiency of intestinal nutrient absorption for proteins, amino acids, and lipids in the intestine modulates muscle growth through insulin, growth hormone, and other key signaling pathways (Figure 4). As a key component of the gut–muscle axis, the intestine and commensal gut microbiota collaboratively regulate the absorption, metabolism and synthesis of dietary nutrients [142,143]. The gut microbiota produces a diverse array of enzymes to degrade dietary fiber, including lignin, non-starch polysaccharides and resistant starch, thereby facilitating nutrient catabolism [142,143]. The microbiota also substantially promotes host nutrient utilization and amino acid biosynthesis, thus providing critical substrate support for muscle protein synthesis [144]. Moreover, the gut microbiota serves as an important regulatory factor, exerting systemic effects on skeletal muscle metabolism via the complex “nutrition intake-signaling system axis” by altering nutrient absorption efficiency and generating metabolic signals [144].

Intestinal absorption of key amino acids, including neutral amino acids and BCAAs, is essential for sustaining plasma and muscular amino acid homeostasis and governing muscle protein turnover [145]. BCAA absorption efficiency tightly controls the balance of muscle protein synthesis and degradation; specifically, leucine modulates muscular mTORC1 activity and protein metabolism via system L transporters [145]. The intestinal absorption of neutral amino acids is primarily mediated by the brush-border transporter neutral amino acid transporter 1 (B0AT1)/solute carrier family 6 member 19 (Slc6a19); dysfunction of this transporter decreases circulating and muscular amino acid availability and impairs muscle protein synthesis [146]. The stability and function of B0AT1/Slc6a19 depend on heterodimerization with angiotensin converting enzyme 2 (ACE2), and deficiency of the ACE2-B0AT1 complex markedly inhibits intestinal neutral amino acid uptake and depletes muscular amino acid substrates [147]. Stress conditions such as chronic cold exposure suppress intestinal BCAA absorption, reduce muscular BCAA levels, and trigger the upregulation of UPS-related atrophy markers MuRF1 and atrogin-1, thereby accelerating muscle protein degradation and atrophy [95]. In contrast, supplementation with rapidly digestible carbohydrates enhances intestinal BCAA uptake, improves muscle amino acid flux, upregulates dystrophin expression, and alleviates muscle protein breakdown [95].

Growth hormone (GH) and insulin-like growth factor 1 (IGF-1) modulate the gut–muscle axis via two pivotal routes: regulating intestinal absorption of amino acids and lipids, and governing skeletal muscle development and systemic nutrient metabolism [148,149]. GH induces IGF-1 production in myocytes to regulate muscle fiber phenotype and growth, and independently enhances skeletal muscle insulin sensitivity in an IGF-1-independent manner [148]. Meanwhile, GH collaborates with insulin to activate the PI3K/Akt/mTOR pathway, which serves as an important signaling node to promote muscle protein synthesis and inhibit protein degradation [148,149]. Additionally, gut microbiota mediates the effects of lifestyle interventions on muscle metabolism [150]. Intermittent fasting enriches *Akkermansia muciniphila*, which inhibits intestinal lipid absorption through the PI3K/Akt pathway, promotes white adipose tissue browning and improves metabolic homeostasis, thereby indirectly influencing muscle energy metabolism and quality maintenance [150]. Intestinal lipid absorption efficiency determines circulating lipid and energy supply; thus, excessive fat absorption under pathological conditions disrupts metabolic homeostasis and indirectly impairs muscle physiological status [151,152]. Furthermore, the probiotic strain TWK10 elevates the abundance of *Desulfovibrionaceae*, *Bifidobacteriaceae*, *Enterobacteriaceae* and *Erysipelotrichaceae*, which are closely correlated with circulating BCAA levels [153]. Such microbial alteration facilitates the production and absorption of EAAs and BCAAs, thereby supporting muscle protein synthesis and muscle strength maintenance [153].

Intestinal nutrient absorption acts as an upstream hub coordinating endocrine hormones and gut microbiota to govern muscle metabolism [148,149]. Absorption efficiency modulates GH and insulin activity as well as downstream anabolic signaling, while microbial metabolism further shapes nutrient availability and systemic metabolic homeostasis [153,154]. As an active regulatory node of the gut–muscle axis, the intestine integrates nutrient sensing, endocrine signaling and microbial cues to modulate muscle quality, providing potential targets for intervening in muscle loss and metabolic disorders.

## 4. Regulatory Mechanisms of FMH Substances in Meat Quality

The gut–muscle axis achieves comprehensive regulation of meat quality by affecting muscle fiber development and transformation, enhancing muscle tenderness and water-holding capacity, promoting flavor deposition, and modulating IMF and fatty acid composition [155]. On this basis, FMH substances mainly improve meat quality through the following four pathways.

### 4.1. Reshaping the Gut Microbiota

Accumulating evidence indicates that certain representative FMH substances are capable of reshaping gut microbiota by enriching beneficial bacteria, suppressing pathogenic taxa, and increasing the production of beneficial microbial metabolites such as SCFAs [156,157]. Such microbial modulation helps establish a balanced intestinal microecology that supports the gut–muscle axis in regulating muscle characteristics and meat quality [156,157]. As pivotal mediators of the gut–muscle axis, SCFAs activate the IGF-1/Akt/mTOR signaling pathway to promote muscle fiber development, increase muscle fiber diameter and cross-sectional area, and ultimately enhance muscle mass [92]. While not classified as an FMH substance, *Clostridium butyricum* has been reported to enhance lamb growth performance and muscle mass via the IGF-1/Akt/mTOR pathway, providing background support for this mechanism [158]. Polysaccharides are the key active components of FMH substances in modulating gut microbiota [19,85]. Astragalus polysaccharides remodel the gut microbial community by enriching *Lactobacillus johnsonii* and *Faecalibaculum rodentium*, promote SCFA production, and improve animal growth performance [85]. Licorice polysaccharides optimize gut microbiota structure by increasing beneficial bacteria (e.g., *Bacteroides*, *Butyricicoccus*, *Eisenbergiella*) and decreasing harmful bacteria (e.g., *Erysipelatoclostridium*) [19,159,160]. Lycium Barbarum polysaccharides have been reported to enhance gut microbiota diversity and modulate the Firmicutes/Bacteroidetes ratio, alongside promoting the proliferation of beneficial bacteria [161]. These changes further exert microbiota-dependent regulation on host metabolism, in part through the enrichment of SCFA-producing taxa within Firmicutes [161]. Ganoderma Lucidum polysaccharides act as fermentation substrates to reduce intestinal pH, increase SCFA levels, and enrich beneficial bacteria, including *Bacteroides*, *Akkermansia*, and *Bifidobacterium* [156].

Other active components of FMH substances also exert gut microbiota-regulating effects linked to muscle and meat quality. Hawthorn pectin functions as a prebiotic by serving as a carbon source for intestinal microbes [162]. Phenolic acids and flavonoids in Hangzhou white chrysanthemum extract increase beneficial bacteria (e.g., *Allobaculum, norank_f__Muribaculaceae*) and improve microbial equilibrium [163]. Lotus seed resistant starch regulates gut microbiota to promote BA metabolism and improve host metabolic homeostasis [164]. Honey increases the abundance of *Muribaculaceae* and reduces serum fatty acids and inflammatory cytokines [70].

**Figure 5:**
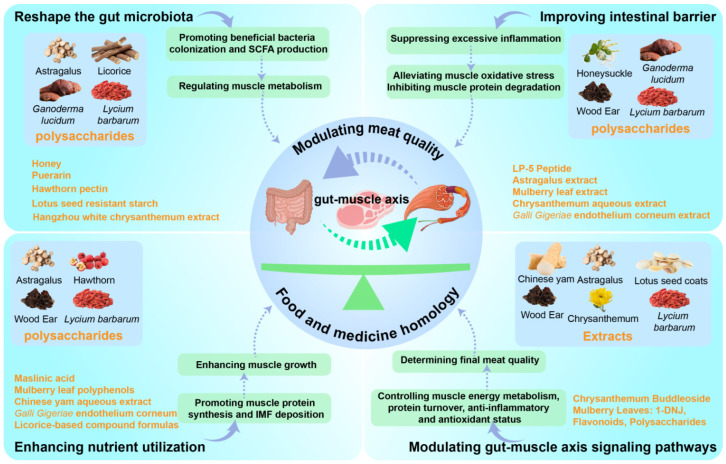
The potential effects of food and medicine homology (FMH) substances on muscle function and meat quality via the gut–muscle axis. Note: Solid lines represent established effects supported by published evidence, while dashed lines indicate hypothetical downstream associations with meat quality. It should be noted that direct validation of these regulatory effects on livestock meat quality remains limited, as most supporting data are derived from non-livestock models.

The optimized gut microbiota and their metabolites can directly or indirectly influence muscle, as shown in Figure 5. For example, puerarin has been shown to modulate gut microbiota to promote SCFA production and enhance ATP synthesis in skeletal muscle, which contributes to the improvement of muscle contractile function and physiological performance [165]. These findings provide indirect mechanistic evidence for muscle physiological regulation. Ganoderma Lucidum promotes Lactobacillus proliferation, enhances microbial diversity, inhibits pathogens such as *Escherichia-Shigella*, and its polysaccharide-derived SCFAs maintain systemic inflammatory homeostasis to support muscle health [156,166]. Lycium Barbarum polysaccharides enrich beneficial microbiota and metabolites to create a favorable internal environment for muscle metabolism and anti-inflammatory processes [52].

In summary, FMH substances modulate the structure and function of gut microbiota, promote beneficial bacteria colonization and SCFA production, and optimize the microbial signal network of the gut–muscle axis, thereby regulating muscle metabolism, inhibiting inflammatory damage, and improving meat quality.

### 4.2. Improving the Intestinal Barrier

Certain representative FMH substances have shown potential to prevent intestinal hyperpermeability and accompanying chronic low-grade inflammation, via strengthening intestinal barrier integrity and suppressing local as well as systemic inflammatory responses [159,167,168]. This reduces inflammation-induced muscle damage and creates a favorable internal environment for muscle growth and health, as summarized in Figure 5. Impaired intestinal barrier function often leads to endotoxin translocation and systemic inflammation, which accelerate muscle protein breakdown, reduce water-holding capacity, and ultimately impair meat quality [155]. Therefore, maintaining intestinal barrier integrity and immune homeostasis is essential for normal muscle development and high-quality meat production [155]. FMH substances directly strengthen the intestinal physical barrier and reduce intestinal permeability, which mechanistically supports their potential benefits to muscle and meat quality [167,168]. Glycyrrhizin from licorice is metabolized by gut microbiota into absorbable active compounds to exert barrier-protective effects [167]. Honeysuckle polysaccharides promote the proliferation of *Bifidobacterium* and *Lactobacillus*, significantly enhancing intestinal barrier function in mice with colitis [168,169]. Lycium Barbarum polysaccharides upregulate the tight junction protein zonula occludens-1, reduce plasma endotoxin and pro-inflammatory cytokine levels, and synergistically enhance intestinal barrier function via regulating butyrate-producing microbiota [52,161,170]. Ganoderma Lucidum polysaccharides maintain tight junction protein expression and intestinal barrier integrity [171]. The pentapeptide LP-5, naturally identified in Galli Gigeriae Endothelium Corneum, directly promotes tight junction protein expression by activating the AhR pathway, while inhibiting NF-κB and c-Jun N-terminal kinase (JNK) inflammatory signaling pathways, thereby facilitating intestinal barrier repair [172,173].

FMH substances inhibit intestinal inflammation and reduce systemic pro-inflammatory factor release, which may indirectly relieve muscle inflammation and protein catabolism, offering mechanistic clues for meat quality improvement [172,174]. Astragalus extract alleviates intestinal inflammation, promotes barrier repair, and rebalances gut microbiota and SCFA/BA metabolism [174]. Galli Gigerii Endothelium Corneum extract inhibits key pro-inflammatory factors, including TNF-α, IL-1β, and IL-6 [172]. *Auricularia* polysaccharides exert anti-inflammatory effects by inhibiting classical pro-inflammatory pathways such as TLR4/JNK and activating the Akt/AMPK pathway, thereby ameliorating obesity and inflammation [175,176]. Mulberry leaf extract enriches *Akkermansia* and *Bifidobacterium*, modulates SCFA metabolism, repairs the intestinal barrier, and reduces endotoxin translocation into the bloodstream [157,177,178]. *Auricularia* polysaccharides improve the intestinal microecology, promote beneficial bacteria and SCFA production, while simultaneously enhancing barrier function and suppressing inflammation [175,176]. *Chrysanthemum morifolium* aqueous extract regulates gut microbiota structure and activates the 15d-PGJ_2_/PPARγ and PPARα pathways to alleviate inflammation and oxidative stress [18].

In summary, FMH substances reinforce the intestinal barrier, suppress excessive inflammation, and optimize gut microbiota, forming a coordinated intestinal defense system in preclinical models. These effects reduce endotoxin translocation and systemic inflammatory burden, which mechanistically supports the potential to alleviate muscle oxidative stress, inhibit muscle protein degradation, and improve meat quality traits such as water-holding capacity and tenderness. However, direct validation of these benefits in livestock feeding trials with meat-quality endpoints remains limited.

### 4.3. Enhancing Nutrient Utilization

FMH substances enhance nutrient digestion, absorption, and utilization by regulating the digestive system, gut microbiota, and intestinal barrier [21,74]. This provides sufficient substrates and energy for muscle growth, IMF deposition, and muscle protein synthesis, which may favorably modulate final meat quality traits (Figure 5).

IMF deposition, collagen characteristics, connective tissue solubility, and calpain-mediated postmortem proteolysis collectively determine meat tenderness, juiciness, flavor and overall sensory quality [179,180,181]. Adequate nutrient supply and efficient utilization are essential for IMF accumulation and normal muscle tissue development [181,182]. FMH substances improve nutrient utilization to support muscle growth and meat quality formation [182,183]. Mulberry leaf polyphenols suppress lipase activity and modulate dietary fat absorption [182], and the subsequent effect on IMF deposition varies with energy balance, animal species and growth stage. Galli Gigeriae Endothelium Corneum improves pancreatic secretion and digestive efficiency, promoting nutrient absorption for muscle anabolism [74]. Chinese yam starch enhances intestinal barrier function and regulates gut microbiota to improve nutrient absorption efficiency [21]. Chinese yam aqueous extract protects gastric tissue integrity and reduces inflammatory injury, creating a healthy environment for nutrient digestion [24]. Maslinic acid derived from hawthorn has been shown to enhance gastric smooth muscle activity in cellular models, which may potentially enhance nutrient digestion efficiency and offer substrate support for muscle development [183].

Certain FMH substances also improve hepatic metabolism and systemic nutrient homeostasis to ensure an efficient supply for muscle synthesis and IMF deposition [184,185]. Astragalus polysaccharide regulates hepatic bile acid metabolism and enhances lipid utilization [186]. Hawthorn polysaccharides and flavonoids modulate gut microbiota, activate AMPK/PPARα pathways, improve hepatic lipid metabolism, and optimize energy supply for muscle and IMF deposition [187,188,189]. Lycium Barbarum polysaccharide regulates glucose and lipid metabolism to enhance nutrient utilization efficiency [50,190,191]. *Auricularia* polysaccharides can alleviate insulin resistance and improve metabolic homeostasis for muscle anabolism [176,192,193]. Rosmarinic acid from mint lowers serum total cholesterol and triglyceride levels in disease model mice, thereby alleviating estrogen deficiency-induced metabolic dysfunction [194]. Although these findings were obtained from disease or non-livestock models, they suggest that FMH substances hold the potential to optimize the systemic distribution of energy and nutrients at the metabolic level.

In summary, FMH substances improve species-specific nutrient digestion, absorption and metabolic utilization via gastrointestinal protection, hepatic function regulation, and gut microbiota modulation. Different FMH ingredients exert regulatory effects through divergent pathways. These physiological changes can provide adequate substrates for muscle protein synthesis and IMF deposition, and thus may contribute to the improvement of meat tenderness, juiciness and flavor.

### 4.4. Modulating Gut–Muscle Axis Signaling Pathways

FMH substances regulate meat quality predominantly via gut-derived signals that target skeletal muscle through the circulation to modulate conserved signaling pathways; however, they can also exert beneficial effects through direct antioxidant activity, antimicrobial activity, modulation of feed palatability and feed intake, as well as direct regulation of muscle metabolism [111,113,174]. These pathways precisely control muscle energy metabolism, protein turnover, anti-inflammatory and antioxidant status, and ultimately determine the physicochemical and eating quality of meat (Figure 5).

Skeletal muscle fiber type composition is a key factor determining meat color; elevated proportions of type I and IIA muscle fibers contribute to improved redness due to higher myoglobin content [181,195]. Myostatin gene deletion reshapes gut microbiota and induces selective hypertrophy of fast-twitch glycolytic muscle fibers, in which the microbial metabolite valerate activates the Akt/mTOR pathway via GPR43 to promote myoblast differentiation and alleviate muscle atrophy [196]. After slaughter, muscle metabolism transitions from aerobic to anaerobic, and meat color is jointly determined by lipid oxidation, myoglobin, metabolites, and mitochondria [197]. Gut microbiota also modulate meat quality by regulating bile acid profiles; for example, high DCA levels are associated with improved muscle fiber development and optimized fat deposition in sheep [93]. Of note, this study provides direct livestock phenotypic evidence linking gut microbial metabolism to meat quality traits. As a typical FMH formulation, huanglian wendan decoction can activate the serine/threonine-protein kinase 11 (STK11, also known as liver kinase B1, LKB1)/AMPK pathway by modulating the gut microbiota and related metabolites, thereby suppressing lipogenesis. Importantly, this activation also helps maintain skeletal muscle contractile function, which in turn contributes to the indirect improvement of meat quality [198,199].

Various FMH active components improve the muscle anabolic environment by regulating energy metabolism and insulin sensitivity-related pathways. Mulberry leaf components (1-deoxynojirimycin, flavonoids, polysaccharides) activate the PI3K/Akt insulin signaling pathway to relieve inflammation and oxidative stress, thereby enhancing insulin sensitivity [200]. PI3K/Akt signaling is a critical switch for muscle protein synthesis and promotes muscle anabolic status [148,149]. Lycium Barbarum aqueous extract maintains skeletal muscle function and inhibits muscle atrophy-related gene expression via pattern recognition receptor signaling [201]. Lotus seed coat phenolic extracts improve systemic glucose and lipid homeostasis, providing a stable energy supply and reducing lipotoxicity in muscle cells [202,203]. Notably, although these hypoglycemic and lipid-regulating effects do not serve as direct evidence for meat quality improvement, such metabolic improvements are mechanistically linked to muscle growth and lipid deposition, and may indirectly modulate meat quality traits.

Certain FMH substances can also help protect muscle health by regulating pathways related to inflammation and oxidative stress [201,204]. Lycium Barbarum aqueous extract alleviates muscle oxidative stress and inflammation in experimental aging animal models [201]. Chrysanthemum-derived buddleoside regulates macrophage balance and inhibits the NF-κB pathway, which blocks inflammation-induced muscle protein breakdown and atrophy [204,205]. These findings are derived from non-livestock animal models and provide indirect mechanistic support for muscle quality regulation.

Finally, FMH substances also indirectly regulate muscle metabolism by remodeling gut-derived metabolic signaling [94,174]. Astragalus extract rebalances gut microbiota and normalizes SCFA and BA metabolism [174]. SCFAs and BAs act as signaling molecules to activate G protein-coupled receptors and the FXR in muscle, thereby modulating energy metabolism and inflammation to maintain muscle homeostasis [94,111,113]. Chrysanthemum aqueous extract activates the PPAR pathway, which regulates muscle fatty acid oxidation and fiber type composition, potentially influencing meat quality characteristics [204,205]. Collectively, these metabolic and pathway regulatory effects represent underlying indirect mechanisms that mediate FMH-induced meat quality improvement.

## 5. Application of FMH in Animal Production

**Figure 6:**
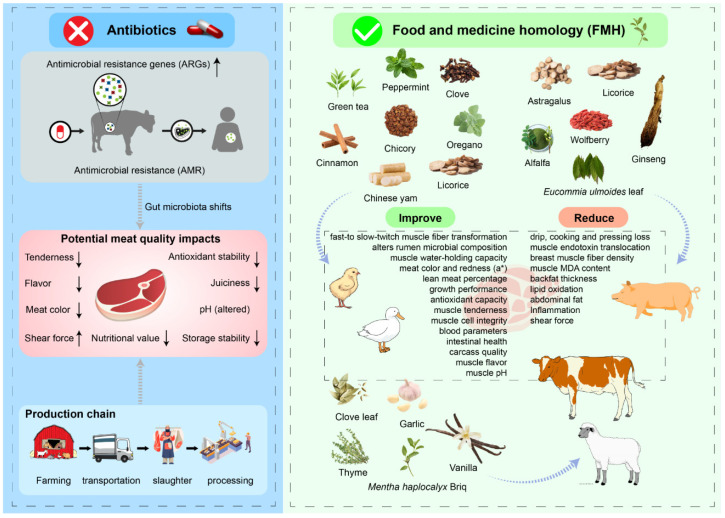
Potential effects of antibiotics and food and medicine homology (FMH) substances on livestock production and meat quality. Note: Dashed lines indicate indirect, context-dependent associations. Antibiotic use is associated with two distinct concerns: public health risks (antimicrobial resistance and residues) and potential alterations in meat quality, which are context-dependent and mediated by secondary factors such as gut microbiota shifts and production conditions.

Meat production constitutes a key part of the global food supply and economy, and consumer preference largely depends on meat quality [206]. High-density farming reduces the accumulation of muscle flavor precursors by changing fatty acid profiles and inhibiting the synthesis of IMF and flavor-related amino acids, thereby diminishing meat juiciness and flavor [207,208]. Oxidative damage during rearing, transport, slaughter, and processing impairs meat quality, causing deterioration in meat color, texture, accelerated spoilage, nutrient loss, and toxic compound formation (Figure 6) [209]. Overuse of antibiotics in conventional production increases antimicrobial resistance genes in animals and the environment, and compromises meat safety and quality, which represents a typical “One Health” issue [210].

As substances with dual edible and medicinal properties, as defined by the concept of food-medicine homology, FMH materials are distinct from probiotics, and there are conventional feed additives in their origin and functional positioning [6,84]. As natural and safe alternatives, FMH substances improve meat quality mainly through the gut–muscle axis by regulating gut microbiota, enhancing intestinal barrier function, promoting nutrient absorption, and modulating key signaling pathways [21,74,200]. This section summarizes the applications of FMH substances in poultry and livestock (Table 2), aiming to provide evidence for developing targeted feed additives and sustainable husbandry strategies.

### 5.1. Poultry

Poultry meat (chicken, duck, goose), especially skinless breast muscle, is characterized by high protein, relatively low calorie, fat and cholesterol levels, as well as high digestibility, which aligns with modern healthy dietary preferences [218,219]. Its market demand is continuously growing due to the advantages of economic affordability, stable quality and diverse culinary applications [220]. As a typical high-quality lean meat, chicken breast is particularly favored for its low-fat and high-protein nutritional profile [220]. Multiple studies suggest that FMH substances may exert favorable effects on poultry meat quality, potentially through modulating muscle fiber traits, elevating antioxidant status, regulating lipid metabolism and improving meat water-holding capacity [211,221,222].

#### 5.1.1. Broiler

IMF is a core evaluation index of broiler meat quality. Moderate and optimal IMF deposition can effectively improve meat juiciness, tenderness and flavor; however, excessive fat accumulation, unfavorable alterations in fatty acid composition, and lipid oxidation will conversely impair muscle physiological function and overall meat quality [223,224]. FMH substances exert tissue-specific regulatory effects on lipid metabolism by modulating the expression of lipid metabolism-related genes, thereby optimizing fat distribution and improving the overall quality and flavor of broiler meat [211]. As a classic FMH-derived active component, epigallocatechin gallate from green tea regulates hepatic lipid metabolism, alleviates inflammatory responses and oxidative damage, and promotes the accumulation of flavor-related amino acids in muscle, thereby protecting flavor precursors and enhancing meat flavor characteristics [211,222]. Curcuminoids, another group of functional FMH plant components, inhibit pancreatic lipase activity to reduce dietary fat absorption and further modulate systemic lipid deposition and metabolism [221]. Polysaccharides from FMH plants regulate lipid metabolism homeostasis, appetite and energy metabolism via the gut–liver axis, and alleviate intestinal oxidative stress and inflammatory injury by reshaping gut microbiota structure [225].

Antibiotic abuse in broiler production disrupts intestinal microecological balance, induces abnormal abdominal fat deposition, and compromises meat quality [226]. Gut microbiota and its metabolites (including SCFAs and BAs) regulate host lipid metabolism signaling pathways, forming a critical “gut microbiota/metabolites-IMF axis” that governs broiler meat quality formation [227]. FMH substances target the “microbiota–metabolite–host” axis to regulate intestinal microecology and microbial metabolite signaling, thereby reprogramming nutrient metabolism, immune response and stress tolerance in broilers and ultimately improving meat tenderness, flavor, juiciness, color and safety [14,131,211]. The combined application of peppermint oil and clove oil, as essential oil-type FMH substances, improves broiler growth performance and carcass traits, reduces abdominal fat deposition and increases lean meat percentage [14]. Such additives optimize blood biochemical parameters and intestinal health by reshaping gut microbial community structure, inhibiting harmful bacteria and enriching beneficial microbes such as Lactobacillus [14]. Likewise, green tea-derived epigallocatechin gallate modulates broiler gut microbial composition by altering the relative abundance of core microbial phyla and genera, further stabilizing intestinal microecological homeostasis [211]. Compound plant-derived FMH extracts from oregano, clove, and cinnamon strengthen intestinal barrier integrity, mitigate intestinal inflammation, and improve nutrient digestibility and growth performance in broilers [131]. Meanwhile, this extract blend maintains muscle cell integrity and activates antioxidant systems, reducing meat drip loss and preserving meat quality [131]. Multiple polysaccharide-based FMH ingredients also exhibit positive regulatory effects on broiler meat quality [16,212]. Chinese yam polysaccharides enhance antioxidant capacity, optimize carcass performance and meat color, and reduce meat shear force [16]. Additionally, licorice polysaccharide acts through a distinct mechanism. At a supplementation level of 1500 mg/kg, it increases breast muscle fiber density, reduces cooking loss and drip loss, and regulates the expression of MyoG and MyoD to improve meat quality [212].

#### 5.1.2. Duck

Shear force is a key indicator of meat tenderness and is widely used to predict consumer acceptance [213,228]. Lower shear force values are associated with finer muscle fibers and higher moisture content, both of which contribute to improved meat quality [228]. For example, as an FMH substance, dietary supplementation with chicory-derived inulin at 20 g/kg has been shown to promote the transformation of duck muscle fibers from the fast-twitch (type II) to the slow-twitch (type I) phenotype. This shift results in reduced shear force and pressing loss in the breast muscle, alongside increased redness and pH values [213]. Furthermore, inulin supplementation enhances antioxidant capacity, retards lipid oxidation, elevates the levels of umami and sweet amino acids, and decreases the abundance of off-flavor compounds [213]. Collectively, these changes lead to a marked improvement in duck meat flavor.

FMH substances achieve multi-dimensional improvements in poultry meat quality by regulating muscle fiber characteristics, lipid metabolism and gut microbiota homeostasis [212,213,227,228]. These findings provide natural regulatory strategies for targeted quality control of poultry products, and further confirm the core role of the gut microbiota–metabolite–host axis in determining meat quality [212,213,227,228]. Future studies should further clarify the molecular mechanisms underlying key active components of FMH substances and explore the synergistic application of multiple FMH ingredients, so as to support the development of green and high-quality poultry production systems.

### 5.2. Livestock

Compared to poultry meat, red meats such as pork, beef, and lamb are valuable sources of dietary protein, EAAs, bioavailable iron and zinc, and B vitamins, occupying an important share in global meat consumption [229]. The redness of meat originates from myoglobin, which contributes to its visual appeal, whereas meat flavor is partially derived from controlled lipid oxidation products such as aldehydes and ketones, and juiciness mainly depends on water-holding capacity and fat content [230]. Although excessive intake of red meat has been associated with certain health risks, moderate consumption of high-quality red meat remains an important component of a balanced diet [231]. Consumer choices for red meat are based not only on its distinctive flavor, juiciness, and satiety but also on a growing emphasis on its nutritional profile [231]. Consequently, improving meat quality, optimizing fatty acid composition, and enhancing the nutritional value of livestock through nutritional strategies have become key focuses of research in animal husbandry and food science. FMH substances, owing to their natural, safe, and dual functional properties in both nutrition and regulation, demonstrate significant potential in enhancing the quality of pork, beef, and lamb.

#### 5.2.1. Pig

Spearman correlation analysis has established an association between specific gut microbiota and pork quality, revealing that unidentified genera within the family *Lachnospiraceae*, together with *Prevotella* and *Alloprevotella*, are positively correlated with IMF content and marbling score in the longissimus dorsi muscle of pigs [206]. Further intervention research and causal validation models are still needed to elucidate their exact regulatory mechanisms. Gut microbiota imbalance reduces the production of beneficial metabolites such as SCFAs and BAs, weakens antioxidant defense capacity, and aggravates lipid peroxidation [11]. Such microbial dysregulation ultimately impairs meat water-holding capacity and increases drip loss [11].

Multiple plant-derived FMH substances, including alfalfa, licorice, goji berry, ginseng, and astragalus polysaccharides, can effectively alleviate the above adverse outcomes by regulating pig intestinal microecology through multiple pathways [214]. These FMH polysaccharides exert potential antibiotic-alternative effects by maintaining intestinal physiological homeostasis, restoring intestinal barrier integrity, and rebalancing gut microbial community structure [214]. They further upregulate tight junction proteins to protect intestinal mucosal integrity and inhibit the secretion of pro-inflammatory cytokines such as TNF-α and IL-6, thereby reducing endotoxin translocation and muscular malondialdehyde (MDA) accumulation, and maintaining pig growth performance, immune function and intestinal health [214]. As crucial FMH bioactive components, protocatechuic acid derived from Eucommia Ulmoides can relieve intestinal oxidative stress and inflammatory damage in pigs [215]. It strengthens intestinal barrier function by modulating tight junction proteins and remodels gut microbiota to a homeostatic phenotype, suppressing pro-inflammatory microbial taxa while enriching anti-inflammatory commensal bacteria [215]. Similarly, licorice extract, a typical FMH additive, ameliorates oxidative stress and improves nutrient absorption in piglets via modulating beneficial intestinal microbial populations [29]. Apart from the intestinal health benefits observed in piglets and growing pigs, independent feeding trials in finishing pigs further confirm that FMH substances can improve pork quality at slaughter [15]. For instance, Eucommia Ulmoides leaf extract (ELE) supplementation exhibits negligible effects on pig growth performance but effectively modulates fat deposition and improves *Longissimus dorsi* muscle properties, including pH, meat color, lightness, and water-holding capacity [15]. Concurrently, FMH intervention remodels fatty acid composition, enriches flavor-related compounds and sweet-tasting amino acids, and boosts antioxidant enzyme activities in both serum and muscle, ultimately suppressing muscular lipid oxidation [15].

Overall, FMH substances may indirectly contribute to pork quality improvement via regulating intestinal microecology, restoring intestinal barrier integrity, modulating host metabolism, and alleviating inflammation and oxidative stress.

#### 5.2.2. Ruminants (Sheep and Cattle)

Ruminants differ from monogastric animals in their digestive physiology, as rumen fermentation, volatile fatty acid metabolism, and microbial protein synthesis jointly shape their unique gut–muscle axis function [216,232]. In finishing sheep, dietary Mentha Haplocalyx Briq (MHB) improves small intestinal villus structure and large intestinal mucosal morphology, and modulates rumen microbiota to enhance nutrient digestion [216]. Similarly, garlic peel supplementation remodels rumen microbial composition and fermentation characteristics, promoting the proliferation of beneficial microbes and inhibiting harmful taxa, thereby improving the growth performance of lambs [232]. Beyond growth regulation, FMH substances modulate ruminant meat quality via the gut microbiota, microbial metabolites, and host muscle metabolism, exerting a key regulatory influence on IMF deposition [217,232].

IMF content and fatty acid profile are key determinants of the sensory quality and nutritional value of ruminant meat, and targeted modulation of IMF deposition effectively improves meat juiciness, tenderness, and flavor [217]. In young bulls, compound FMH additive intervention significantly improves multiple meat quality traits, including muscle pH, shear force, and oxidative stability, and enhances the sensory acceptability of meat tenderness [217].

Overall, FMH substances may help optimize rumen-intestinal microecological homeostasis, potentially improve the host’s metabolic and antioxidant capacity, and thereby contribute to the improvement of carcass performance, sensory quality, and nutritional value of ruminant meat. This intervention could provide a promising, sustainable green strategy for antibiotic replacement and high-quality ruminant meat production.

## 6. Future Perspectives

Nevertheless, FMH substances are inherently complex, typically comprising multi-component mixtures [17,225]. The dominant active ingredients and their synergistic interactions, guided by TCM principles such as “monarch, minister, assistant, and guide”, remain poorly understood, alongside inconsistent compound standardization across studies [5,17]. Furthermore, dose–response relationships for most FMH interventions are still lacking, and direct meat-quality endpoint trials remain scarce, with much evidence confined to rumen fermentation and growth performance rather than phenotypic meat quality [232]. In addition, the causal validation of the gut–muscle axis regulatory mechanisms remains weak, and long-term safety data across animal species and growth stages are insufficient, further complicating the establishment of unified quality controls and stable application protocols [11]. Key challenges lie in standardizing extract composition, botanical origin, plant part, extraction solvent, purity, polysaccharide molecular weight, active-compound quantification, and batch-to-batch consistency. Other critical concerns include formulation standardization under traditional theoretical guidance, efficacy and safety assessments of complex herbal mixtures, and variability in individual animal responses. Additional feed-related risks also need attention, such as maximum dietary inclusion levels, palatability and feed intake performance, anti-nutritional factors, herb–drug interactions, residual hazards, withdrawal period arrangement, and regulatory compliance. These unresolved gaps hinder the large-scale standardized application of FMH in commercial animal farming.

Future research should adopt multi-omics strategies to further elucidate the molecular interaction mechanisms among FMH active components, gut microbiota and host muscle metabolism. Further causal verification is highly warranted to unravel the regulatory interplay within this axis, which can be accomplished by employing representative methodological frameworks, including fecal microbiota transplantation, germ-free or antibiotic-depleted animal models, targeted receptor inhibition, isotope tracing of microbial metabolites, and systematic integrated validation combining microbiome, metabolome and transcriptome profiling. Optimizing ingredient extraction, modification and delivery technologies will help improve the stability and bioavailability of FMH substances. Moreover, establishing species-specific application schemes and unified quality evaluation standards is essential to promote the transformation of FMH technology from laboratory research to practical antibiotic-free livestock production, so as to support the sustainable development of high-quality and safe animal production [233].

## 7. Conclusions

FMH substances exhibit significant potential for advancing environmentally friendly animal husbandry. They may exert effects via the gut–muscle axis, with possible improvements in gut health, nutrient utilization and IMF deposition. These modulations could further affect meat flavor, tenderness, juiciness and nutritional properties. Additionally, FMH-related approaches hold potential to lower dependence on antibiotics and synthetic additives, which may help elevate food safety standards.

In summary, FMH substances connect traditional medicinal resources with modern animal production, and appear capable of modulating the gut–muscle axis to potentially enhance meat quality and production sustainability. Nevertheless, relevant deficiencies still exist, including unclear dose–response patterns, limited direct phenotypic trials, insufficient causal verification data, unassessed long-term safety risks and imperfect application standardization. Further mechanistic research and technical refinement are still required. With continuous exploration, FMH-based strategies may support the development of antibiotic-free and high-standard livestock breeding and offer novel references for sustainable animal husbandry.

## Figures and Tables

**Table 1:** Classification, main chemical components and pharmacological effects of common FMH substances.

Source	Name of TCM in Chinese	Name of TCM in English (Latin)	TCM Properties	Characteristic Chemical Composition	Pharmacological Effects	References
Plant-derived FMH	Shanyao	Chinese yam (Dioscoreae Rhizoma)	Tonify spleen-stomach, nourish lung and kidney, consolidate essence	Polysaccharides, allantoin; fatty acids, amino acids and proteins	Immunomodulatory, antioxidant, anti-aging, anti-tumor, hypoglycemic, gastrointestinal protection	[21,22,23,24,25,26]
	Gancao	Licorice (Glycyrrhizae Radix et Rhizoma)	Tonify spleen and replenish qi, clear heat and detoxify, resolve phlegm to relieve cough	Triterpenoids (glycyrrhizic acid, glycyrrhetinic acid), flavonoids	Anti-inflammatory, antioxidant, antiviral, immunomodulatory	[27,28,29,30]
	Huangqi	Astragalus (Astragali Radix)	Tonify qi and consolidate exterior, promote diuresis, detoxify and heal sores	Triterpenoid saponins, polysaccharides, flavonoids	Immunomodulatory, hypoglycemic, antioxidant, anti-inflammatory, antiviral, antitumor, cardioprotective, regulates gut microbiota	[31]
	Bohe	Wild mint Herb (Menthae Haplocalycis Herba)	Disperse wind-heat, clear head and eyes, soothe throat, regulate liver qi	Essential oils, flavonoids, phenolic lignans, stilbenes	Antioxidant, antibacterial, anti-inflammatory, regulates gastrointestinal function	[32,33,34]
	Sangye	Mulberry leaf (Mori Folium)	Disperse wind-heat, clear liver fire, moisten lung dryness	Polysaccharides, polyphenols, alkaloids; dietary nutrients	Antidiabetic, antioxidant, anti-inflammatory	[35,36,37,38,39,40,41]
	Jinyinhua	Japanese honeysuckle flower bud (Lonicerae Japonicae Flos)	Clear heat and detoxify, disperse wind-heat	Iridoids, organic acids, flavonoids, polysaccharides	Anti-inflammatory, antibacterial, antioxidant, immunomodulatory, antidiabetic, antitumor	[42,43,44,45]
	Juhua	Chrysanthemum (Chrysanthemi Flos)	Disperse wind-heat, clear liver to improve vision	Flavonoids, caffeoylquinic acids, terpenoids	Antioxidant, anti-inflammatory, neuroprotective	[46,47,48,49]
	Gouqizi	Wolfberry (Lycii Fructus)	Tonify liver and kidney, nourish essence and blood	Functional amino acids, unsaturated fatty acids, vitamins and mineral elements	Immunomodulatory, antioxidant, antitumor, anti-inflammatory, hepatorenal protective	[50,51,52]
	Shanzha	Chinese hawthorn fruit (Crataegi Fructus)	Promote digestion and remove stagnation, regulate qi and dissipate blood stasis	Polyphenols, flavonoids, triterpenoids; pectin and choline	Promotes digestion, antioxidant, anti-inflammatory, cardiovascular protective	[53,54,55,56]
	Xingren	Ansu apricot seed (Armeniacae Semen Amarum)	Relieve cough and asthma, moisten intestines to relax bowels	Polyphenolic compounds, sulfur-containing amino acids, lipids and fiber	Antioxidant, antibacterial, anti-inflammatory	[57,58,59]
	Lianzi	Lotus seed (Nelumbinis Semen)	Strengthen spleen to stop diarrhea, tonify kidney to consolidate essence, nourish heart and calm mind	Proanthocyanidins, flavonoids, alkaloids, amino acids	Anti-inflammatory, antitumor, detoxification, cardiovascular protective, antioxidant	[60,61,62,63]
Animal-derived FMH	Fengmi	Honey (Mel)	Clear heat, tonify the middle energizer, and detoxify	Glucose, fructose, phenolics, amino acids and proteins	Antibacterial, anti-inflammatory, antioxidant, modulates blood lipids, modulates gut microbiota	[64,65,66,67,68,69,70]
	E’jiao	Ass-hide gelatin (Asini Corii Colla)	Nourish blood and yin, moisten dryness, and stop bleeding	Collagen hydrolysates, glycosaminoglycans, trace elements	Blood tonification, immune regulation, antibacterial, antioxidant	[71,72,73]
	Jineijin	Chicken’s gizzard membrane (Galli Gigerii Endothelium Corneum)	Promote digestion and invigorate stomach, relieve strangury and dissolve stones	Bioactive peptides, enzymes, amino acids and trace elements	Promotes digestion, regulates glucose metabolism, anti-inflammatory, dissolves stones	[74,75]
Fungus-derived FMH	Lingzhi	Reishi Mushroom (Ganoderma)	Tonify qi, calm mind, relieve cough and asthma, strengthen body resistance	Ganoderma lucidum polysaccharides, ganoderic acids	Antitumor, antioxidant, anti-inflammatory, immunomodulatory, regulates gut microecological balance	[8,76,77]
	Muer	Wood Ear (Auricularia)	Nourish yin and moisten dryness, promote defecation and enrich blood	Polysaccharides, melanin, polyphenols	Antioxidant, anti-biofilm, hepatoprotective	[78,79,80,81,82]

Note: Traditional Chinese Medicine (TCM) terminology used in Table 1 is defined as follows: Tonify: To replenish physical deficiency and strengthen the physiological functions of internal organs (e.g., spleen–stomach, liver, lung and kidney). Consolidate essence: To stabilize physical vitality and improve overall bodily constitution. Tonify qi (also Replenish qi): To boost physical vitality and strengthen the body’s defense capacity. Clear heat: To reduce internal inflammatory tendency and eliminate metabolic heat accumulation. Wind-heat: A common exogenous pathogenic factor that causes fever, sore throat and mild respiratory symptoms. Regulate liver qi: To ease hepatic functional stagnation and enhance systemic physiological circulation. Clear liver fire: To reduce hepatic functional hyperactivity and relieve related physical discomforts. Moisten dryness: To reduce pulmonary internal heat and moisten dry respiratory tissues. Nourish essence and blood: To replenish nutritional deficiency and support normal hematopoietic function. Remove stagnation: To ease gastrointestinal retention and relieve visceral functional stagnation. Dissipate blood stasis: To improve microcirculation and remove local blood flow obstruction. Nourish heart: To support cardiac physiological function and relieve mental tension and restlessness. Tonify the middle energizer: To strengthen gastrointestinal digestion and nutrient absorption function. Relieve strangury: To ease urinary discomfort, including difficult and painful urination.

**Table 2:** The application effects of FMH substances on improving meat quality of poultry and livestock.

FMH Substances	Active Components	Animal	Dosage	Action Pathways	Meat Quality Improvement Effect	Reference
Green tea	Epigallocatechin gallate	Broiler	750 mg/kg diet	Direct	Increases muscle flavor-related amino acids, enhances antioxidant capacity to reduce lipid oxidation, and improves muscle flavor.	[211]
Peppermint and Clove	Mixed essential oils	Broiler	300 mg/kg diet	Indirect	Improves growth and carcass quality (reduced abdominal fat, increased lean meat percentage); modulates gut microbiota to benefit blood parameters and intestinal health.	[14]
Oregano, Clove, and Cinnamon	Synergistic plant extract blend (Fytera Perform)	Broiler	25 g/t diet	Direct + Indirect	Reduces drip loss, enhances water-holding capacity, and improves meat quality by regulating muscle cell integrity and antioxidant pathways.	[131]
Chinese yam	Polysaccharide	Broiler	500 mg/kg diet	Direct	Enhances carcass performance, meat color, reduces shear force, and elevates antioxidant capacity.	[16]
Licorice	Polysaccharide	Broiler	1500 mg/kg diet	Direct	Increases breast muscle fiber density, reduces cooking and drip loss; modulates MyoG/MyoD mRNA expression to improve tenderness.	[212]
Chicory	Inulin	Duck	20 g/kg diet	Direct + Indirect	Promotes fast-to slow-twitch muscle fiber transformation; reduces shear/pressing loss, increases pH/redness (a*), inhibits lipid oxidation, and elevates umami/sweet amino acids.	[213]
Alfalfa, Licorice, Wolfberry, Ginseng, Astragalus	Polysaccharide	Pig	—	Indirect	Reduces muscle endotoxin translocation and malondialdehyde (MDA) content; supports growth, immunity and gut health, thereby benefiting the regulation of meat quality.	[214]
Eucommia Ulmoides	Protocatechuic acid	Pig	4000 mg/kg diet	Indirect	Alleviates oxidative stress, inflammation and intestinal barrier dysfunction by upregulating tight junction proteins and rebalancing gut microbiota.	[215]
Licorice	Extract	Piglets	400 g/t diet	Indirect	Alleviates oxidative stress and improves nutrient absorption by enriching beneficial gut bacteria (Rikenellaceae_RC9_gut_group).	[29]
Eucommia Ulmoides leaf	Extract	Pig	0.2% diet	Direct	Reduces backfat thickness; improves longissimus dorsi muscle pH/meat color, decreases L* and fluid losses; optimizes fatty acid profile and increases flavor compounds (inosinic acid, amino acids); reduces MDA content.	[15]
*Mentha haplocalyx* Briq	mint powder	Finishing sheep	80 g/kg diet	Indirect	Increases small intestinal villus thickness, promotes large intestinal mucosal development, and optimizes rumen microbial structure to improve digestive function.	[216]
Garlic	Peel extract	Sheep	80 g/kg diet	Indirect	Alters rumen microbial composition and fermentation; promotes beneficial genera growth and reduces Fretibacterium to improve lamb growth performance.	
Clove leaf, Vanilla, Thyme	Clove leaf essential oil, vanillin-eugenol-thymol blend, castor oil, cashew oil	Young bulls	3 g/animal/day in diet	Direct + Indirect	Improves meat pH, shear force and oxidative stability; enhances consumer acceptability of tenderness.	[217]

Note: Direct: Studies that directly measured meat quality-related indicators, muscle fiber traits, muscle antioxidant status and muscle gene expression. Indirect: Studies that did not directly measure meat quality, but assessed effects via gut microbiota, intestinal function, blood physiology and growth performance. Direct + Indirect: Studies that evaluated both direct meat quality parameters and indirect physiological indices. a*: CIE color parameter indicating redness (positive value = red). L*: CIE color parameter indicating lightness (0 = black, 100 = white).

## Data Availability

No new data were created or analyzed in this study.

## Abbreviations

Akt, protein kinase B; AMPK, AMP-activated protein kinase; ACE2, angiotensin-converting enzyme 2; B0AT1, neutral amino acid transporter 1; BAs, bile acids; BCAAs, branched-chain amino acids; CA, cholic acid; DCA, deoxycholic acid; LCA, lithocholic acid; EAAs, essential amino acids; FoxO3a, Forkhead box O3a; GPR43, G protein-coupled receptor 43; IGF-1, insulin-like growth factor 1; IMF, intramuscular fat; IL, interleukin; IPA, indole-3-propionic acid; IR, insulin receptor; LPS, lipopolysaccharides; MDA, malondialdehyde; MHB, Mentha haplocalyx Briq; MuRF1, muscle RING-finger protein-1; mTOR, mechanistic target of rapamycin; mTORC1, mechanistic target of rapamycin complex 1; MyoD, myoblast determination protein 1; MyoG, myogenin; MyH, myosin heavy chain; NAFLD, non-alcoholic fatty liver disease; NF-κB, nuclear factor kappa-B; NLRP3, NOD-like receptor family pyrin domain-containing 3; PAMPs, pathogen-associated molecular patterns; PI3K, phosphatidylinositol 3-kinase; PPAR, peroxisome proliferator-activated receptor; SCFAs, short-chain fatty acids; SMOX, spermine oxidase; S6K1, p70 ribosomal protein S6 kinase 1; STK11, serine/threonine-protein kinase 11, also known as LKB1; TNF-α, tumor necrosis factor-alpha; TLR4, Toll-like receptor 4; Treg, regulatory T cell; TGR5, G protein-coupled bile acid receptor 1 (also known as GPBAR1); TWK10, TWK10 probiotic; TCM, traditional Chinese medicine; FMH, food and medicine homology; JNK, c-Jun N-terminal kinase; Slc6a19, solute carrier family 6 member 19.
